# Neuro-Pathology of Intermittent Fever

**Published:** 1856-05

**Authors:** G. W. Hall

**Affiliations:** Bainbridge, Indiana


ARTICLE III.—*Neuro-Pathology of Intermittent Fever.* By G. W. HALL, M.D., of Bainbridge, Indiana.

Some pathologists assert that all diseases commence by disturbance of the function of the solid parts of the machine, and first of all of the nervous system. This view of the origin of, and seat of disease, appears to be very plausible and correct, since the nervous system is so predominant, and regulates and supplies all with energy. The disease that I would here introduce as a representative of those thus originating, or in other words, chiefly implicating the nervous system, is intermittent fever. The poison which produces this disease, after gaining access into the system, which it may do by being inhaled into the lungs, and there being in contact with the interior of the air cells, passes through and enters the circulation, where it may be distributed to the entire system, thereby deranging the nervous system by coming in contact with the extremities of the nerves; or the poison may act by making its first impression on the nerves in the lungs, whence it may travel through the entire nervous system, as electricity does a wire. As to the character or nature of the poison, all that we know is certainly hypothetical. It may enter the system in a gaseous form, which being sedative, or narcotico-irritative, produces by its effects on the nervous system a state of depression, or the first stage of the fever, which is soon followed by reaction; be this as it may, the nervous system appears to bear the onus of disease, the different organs of the body becoming deranged in function, as they do in neuroses, or other nervous disorders. It is well known that in hysteria and neuralgia, and other nervous diseases, the various organs of the body become deranged. Every observing physician knows the great influence of the nervous system over the circulatory and secretory functions, the modifications of secretion and calorification, which result from varying states of the innervation. We know that in our pernicious or congestive fevers, the nervous system is greatly at fault; all of the alarming symptoms are due to a want of a proper innervation, especially of the extreme vessels, which causes a retreat from the periphery of the body towards the centre, and this fever is nothing but a variety of intermittent, or autumnal fever. Hence, intermittent fever I class among the neuroses. I was led to give it this nosological classification from the therapeutical effects of the remedies prescribed, and the close observance of the effects therefrom. Quinine, arsenic, opium, iron, sulph. copper, strychnine, our chief remedies in intermittent fever. These are also our chief reliance in the treatment of nervous diseases.

In those localities where intermittent fever mostly prevails, we see also many cases of periodical neuralgia, which is connected with, or has its origin in the same cause, and requires the same remedies for its cure. We may sometimes, by watching the effects of our remedies, arrive at the nature of the disease—for instance, we know that opium will relieve the pain in neuralgia, whereas, in that of inflammation, it may aggravate it, while bloodletting will aggravate neuralgia, and relieve inflammation.

The practice which I have found to give speediest relief is, when called to a patient in the exacerbation of fever, with hot skin, frequent pulse, severe pain in the head and back, to an adult I would give 3 to 4 grs. of opium, which in a short time (one or two hours) produces a relaxation of symptoms, and the patient is comparatively easy, then complete the cure with quinine. If there appears to be much biliary derangement, in lieu of the opium I would give ½ gr. morphine, with 10 or 15 grs. calomel, which produces the same mitigation of symptoms as the opium does, completely relieving the severe pains in the head and back; and if in four or six hours the bowels should not act, oil or sulph. magnesia should be given. I have never found the large doses of opium above specified produce any bad effect, even in the most robust and phlethoric individuals; on the other hand, it has never failed to give complete relief, given in exacerbation of the fever, it is always followed by a profuse perspiration, and, of course, relief of the febrile symptoms. 3 grs. of opium with 3 grs. quinine, given two or three hours before the expected chill, will prevent it as completely as three times the quinine without the opium.

I have above hinted at a subject, which I should like to see more thoroughly investigated. I should like to hear from the abler of the profession, especially from Professor N. S. Davis, through the pages of the *Journal.*

